# 2360. Factors Affecting COVID-19 Vaccine Uptake in People Living with HIV

**DOI:** 10.1093/ofid/ofad500.1981

**Published:** 2023-11-27

**Authors:** Victoria N Kunkel, Nathan A Summers, Nupur Singh

**Affiliations:** University of Tennessee Health Science Center, Memphis, Tennessee; University of Tennessee Health Science Center, Memphis, Tennessee; University of Tennessee Health Science Center College of Medicine, Memphis, Tennessee

## Abstract

**Background:**

There is a lack of available data regarding COVID-19 vaccination rates in people living with HIV (PLWH). We examined a group of PLWH to evaluate the association between specific demographics and COVID-19 vaccine uptake.

**Methods:**

This is a retrospective observational study which included PLWH who were seen in an HIV clinic at an academic medical center in Memphis, TN. 300 randomly selected PLWH who had been seen at least once in the clinic within an 18-month study period during the COVID-19 pandemic were included. Univariate analysis using Chi Square and T tests followed by multivariable regression including significant results from univariate analysis identified factors associated with COVID-19 vaccine uptake.

**Results:**

300 PLWH between January 2021 to June 2022 were included in the study. 248 (82.67%) were Black, 27 (9%) were White, 2 (0.67%) were Asian, and 23 (7.67%) were of multiracial/other races. The average age was 47 years old. 243 (81%) were retained in care, defined as 3 visits within the 18-month period with 2 of them being at least 6 months apart. 239 (81.29%) of the participants had a suppressed HIV viral load, defined as HIV RNA < 200 copies/mL. The mean number of total vaccinations in the previous 3 years was 4.61, and the mean number of influenza vaccinations in the past 3 years was 1.64. 207 (69%) of the participants received at least 1 COVID-19 vaccine, with 187 (62.33%) being fully vaccinated, defined as 2 doses of mRNA or 1 adenovirus vector vaccine. Age, number of attended clinic visits, retention in care, viral suppression, and number of previous vaccinations were associated with receiving at least one COVID-19 vaccine in univariate analysis, while only age (odds ratio (OR) 1.07, p< 0.0001), number of influenza vaccinations in the past 3 years (OR 0.58, p=0.03), and total number of vaccinations in the prior 3 years (OR 2.29, p< 0.0001) were significant in multivariable analysis.

**Table 1.**
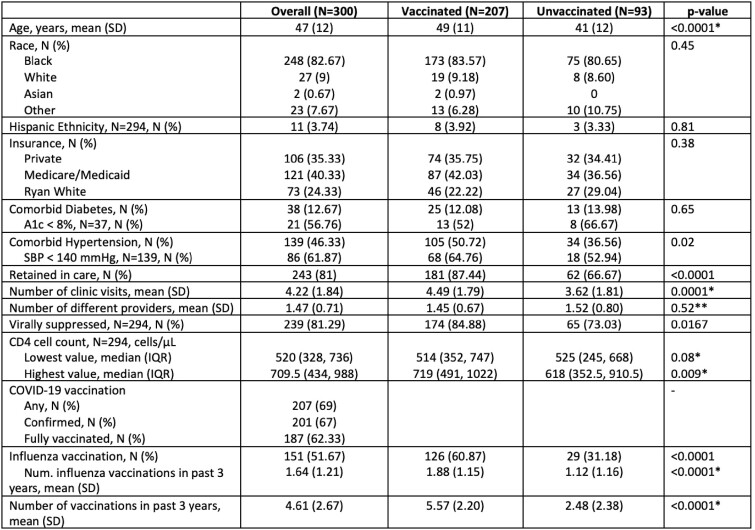
Demographics and COVID-19 vaccine uptake, N=300.

**Table 2.**
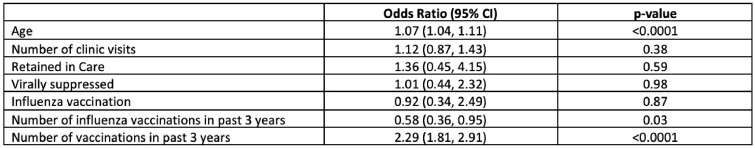
Multivariable analysis, N=294.

**Conclusion:**

Age and total number of vaccinations in the past 3 years were positively correlated with having at least one COVID vaccine. Number of influenza vaccinations in the past 3 years was negatively correlated with having at least one COVID vaccine. Further study is needed to identify targeted interventions to improve vaccine uptake in this population.

**Disclosures:**

**All Authors**: No reported disclosures

